# Supplementary material for: Thermochromic Fibers via Electrospinning
Source: Polymers (Basel). 2020 Apr 6;12(4):842. doi: 10.3390/polym12040842 (PMC7240475; doi:10.3390/polym12040842)
Supplement: Supplementary file 1 [file polymers-12-00842-s001.pdf]

polymers

Supplementary Material

Thermochromic Fibers via Electrospinning

Jimmy Nguyen[†, 1], Ratib M. Stwodah[†, 1], Christopher L. Vasey[1], Briget E. Rabatin[1], Benjamin Atherton[1], Paola A. D'Angelo[2], Kathleen W. Swana[2], and Christina Tang[1*]

[1]Chemical and Life Science Engineering, Virginia Commonwealth University, Richmond, VA 23284 USA; ctang2@vcu.edu, nguyenj28@vcu.edu, stwodahrm@vcu.edu, vaseycl@vcu.edu, athertonbe@vcu.edu, and rabatinb@vcu.edu
[2]U.S. Army Combat Capabilities Development Command Soldier Center, Natick, MA 01760 USA; paola.a.dangelo.civ@mail.mil and kathleen.w.swana.civ@mail.mil

† These authors contributed equally

* Correspondence: ctang2@vcu.edu

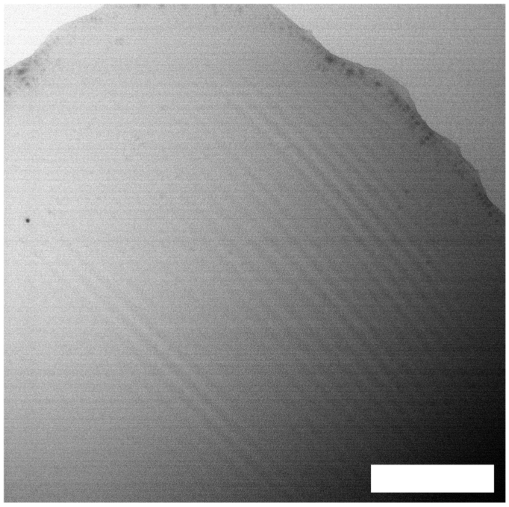

Figure S1. SEM images of electric field applied to 10:1 LC-2:solvent (7:3 toluene:acetone) with no polymer with a 300 micron scale bar. Large droplets were observed and no fibers formed.

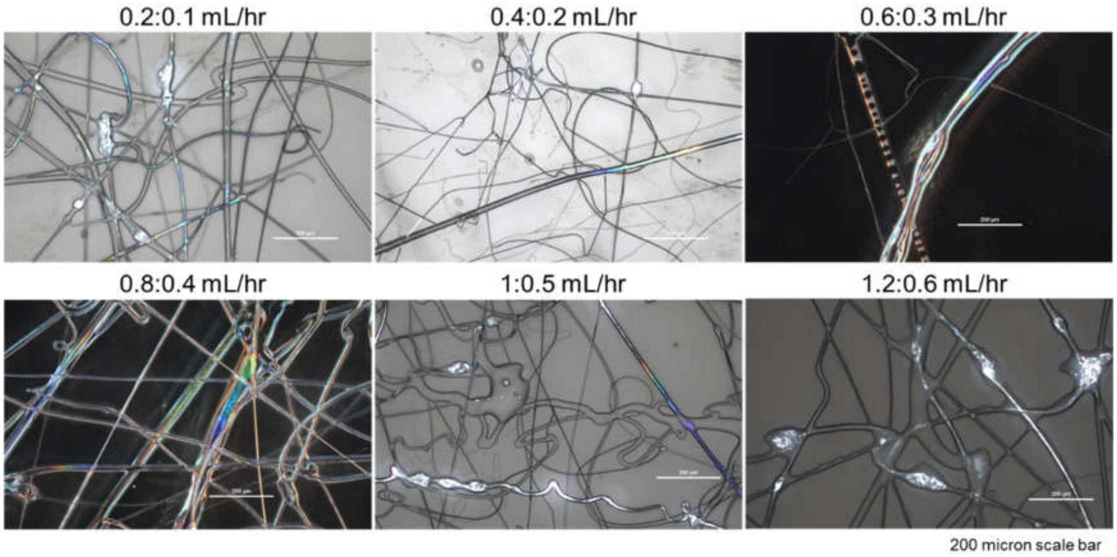

Figure S2. PLM images of coaxial PVP shell, LC-core fibers electrospun at a 2:1 shell:core flow rate ratio at increasing total flow rates. Defects increase as the total flow rate increases.

To increase fiber uniformity, we increased the flow rate ratio of the shell to the core to 4:1. At this ratio, uniform fibers were achieved at throughputs of 0.5 mL/hr and 1 mL/hr, with some bead defects apparent at 1.5 mL/hr (Figure 11).

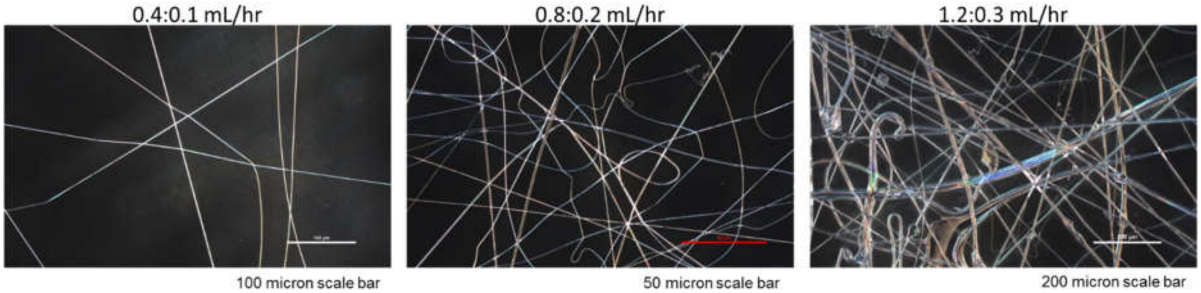

Figure S3. PLM images of coaxial PVP shell, LC-core fibers electrospun at a 4:1 shell:core flow rate ratio at increasing total flow rates. Uniform fibers were obtained at a total throughput of 0.5 and 1 mL/hr. Some bead defects are present at 1.5 mL/hr.

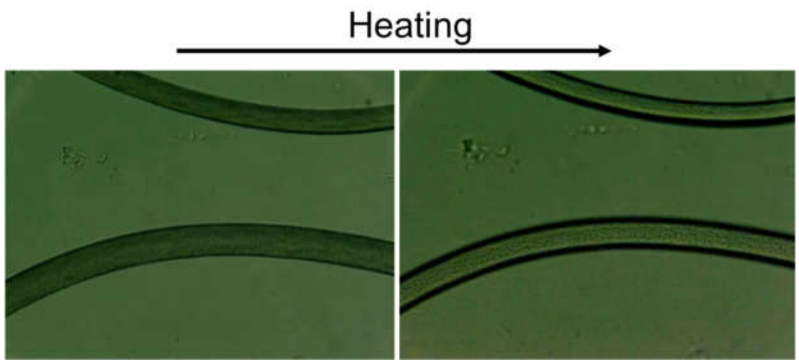

Figure S4. Representative PLM of blend fibers containing LC upon heating the stage from ~25°C to 50°C showing an apparent phase change (20 wt.% PS, 20 wt.% LC-1). No thermochromic behavior is observed.

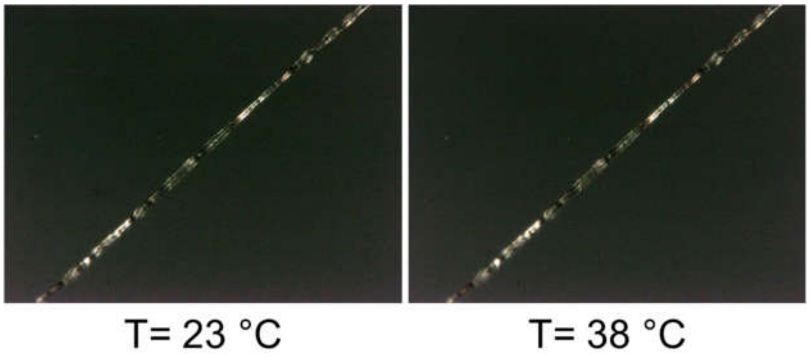

Figure S5. PLM images of polystyrene only fibers (without liquid crystal) heated. No change was observed upon heating.

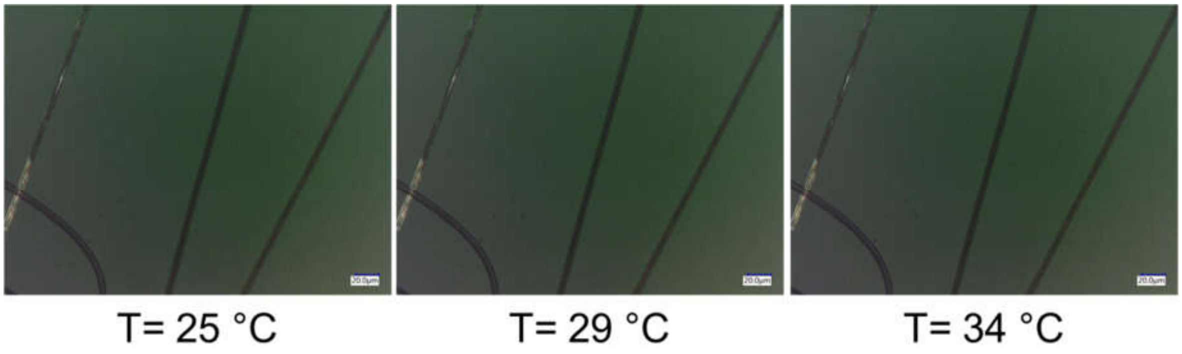

Figure S6. PLM images of coaxial fibers spun from 15 wt.% PVP in ethanol in the shell and 7:3 v:v toluene:acetone in the core at 4:1 shell:core flow rate ratio and a total flow rate ratio of 1 mL/hr. Fibers were heated. No change was observed upon heating.
